# The Value of Disinfectants

**Published:** 1921-11

**Authors:** 

**Affiliations:** Locksley Street, Limehouse, E. 14.


*To the Editor of* THE HOSPITAL AND HEALTH REVIEW

SIR,—Our attention has been drawn to the letter signed by "A Rural Medical Officer" which appears on page 29 of your October issue.

We do not wish to be drawn into a controversy the subject of which has been thrashed out to the satisfaction of scientific authorities in the past, and we enclose herewith for your perusal a letter which appeared in the "Municipal Engineering and Sanitary Record" of the 27th ult. bearing upon the same subject, together with a copy of the article referred to in that letter on "Fumigation as a Disinfecting Agency." We shall be obliged to you if you will kindly make your correspondent acquainted with these matters, and in conclusion we can only express our surprise that any "Rural Medical Officer" could find himself in such a position as to write in the terms he has written on a subject with which he ought to possess personal information.

If need be, facts and figures could be produced from the records of educational authorities to prove the value of disinfectants, and there is the obvious fact of more recent date that during the war something between five and eight million gallons of disinfectant fluid were used by the allied armies under the advice and control of the leading sanitary experts, with the result that the armies were kept practically immune from infectious disease.—Yours faithfully,

THE "SANITAS" COMPANY, LTD.
N. F. KINGZETT, Managing Director.
Locksley Street, Limehouse, E. 14.

